# Genome-Wide Identification of *ATL* Gene Family in Wheat and Their Expression Analysis in Response to Salt Stress

**DOI:** 10.3390/plants14091306

**Published:** 2025-04-25

**Authors:** Xuqing Li, Shuotong Liu, Pei Yu

**Affiliations:** SDU-ANU Joint Science College, Shandong University, Weihai 264209, China; 202200700253@mail.sdu.edu.cn (X.L.); liushuotong@mail.sdu.edu.cn (S.L.)

**Keywords:** Arabidopsis Tóxicos en Levadura, E3 ligases in wheat, whole-genome characterization, abiotic stress

## Abstract

Wheat (*Triticum aestivum*) is one of the most important cereal crops globally, with significant economic value. The Arabidopsis Tóxicos en Levadura (*ATL*) gene family, which comprises members of ubiquitin ligase enzymes (E3s), functions in substrate protein tagging during ubiquitin-mediated protein modification. Recent studies have demonstrated its involvement in stress responses. However, the *ATL* gene family in wheat remains poorly characterized. This study aimed to identify the members of the *ATL* gene family in wheat and investigate their roles under salt stress. We identified 334 *TaATL* genes in the wheat genome, all of which contain either RING-H2, RING U-box, or RAD18 superfamily domains, exhibiting a remarkably low proportion of intron-containing genes. The Ka/Ks (non-synonymous to synonymous substitution rate) analysis and *cis*-acting element analysis of the *TaATL* gene family indicate that its sequences are highly conserved and functionally constrained, suggesting that it may participate in abiotic stress responses through the ABA, MeJA, and MYB signaling pathways. Both RNA-seq analysis and RT-qPCR data demonstrated that the expression levels of the *TaATL* gene family were significantly upregulated under stress conditions, indicating their crucial roles in stress responses. This study demonstrates that the targeted regulation of stress-responsive signaling pathways mediated by superior *TaATL* gene family members can effectively enhance wheat salt tolerance, thereby providing a viable strategy for the development of high-yielding cultivars adapted to saline agricultural ecosystems.

## 1. Introduction

Wheat (*Triticum aestivum*), a pivotal cereal crop in global agriculture, serves as a primary source of dietary calories and protein for a substantial proportion of the world’s population. Nevertheless, wheat productivity is persistently challenged by diverse abiotic stresses, including drought, salinity, extreme thermal fluctuations, and others [1]. These environmental stresses significantly constrain wheat growth and development, ultimately affecting grain yields, thereby posing substantial threats to global food security [2]. Among these, salt stress adversely impacts plant growth and yields by inducing the accumulation of reactive oxygen species (ROS), which subsequently cause oxidative damage to DNA and proteins, disrupting cellular homeostasis and metabolic processes [3]. In the context of the escalating global population and intensifying climate change impacts, which exacerbate the frequency and magnitude of these stresses, elucidating the molecular mechanisms underlying wheat’s response to abiotic stresses has become increasingly imperative [4].

The ubiquitin–proteasome system (UPS) represents a fundamental molecular mechanism in plant stress adaptation, orchestrating the degradation of misfolded or damaged proteins and modulating the turnover of regulatory proteins involved in stress signaling pathways [5]. Central to this system are E3 ubiquitin ligases, which are key determinants of target specificity for degradation by the 26S proteasome system [2,6]. Among these, the Arabidopsis Tóxicos en Levadura (*ATL*) family of RING-type E3 ubiquitin ligases, characterized by the presence of a conserved RING-H2 domain that is essential for their catalytic activity, has emerged as crucial regulators of plant stress responses [7].

The functional significance of *ATL* genes in stress adaptation has been extensively documented across diverse plant species, revealing the evolutionary conservation of their regulatory roles. In the model plant *Arabidopsis thaliana*, multiple *ATL* members have been implicated in mediating responses to various abiotic stresses, including drought, salinity, and cold stress [8]. Notably, in *Oryza sativa*, the *ATL*43 knockout line displays ABA hyposensitivity, suggesting its involvement in stress signaling through the ABA-dependent pathway [8]. Similarly, in maize (*Zea mays*), the expression of *ZmATL10* is significantly upregulated under high-temperature conditions, and the overexpression of this gene enhances thermotolerance, further underscoring the potential regulatory role of *ATL* genes in stress adaptation [9]. These collective findings position *ATL* genes as integral components of the plant stress signaling network, modulating critical physiological and biochemical processes to enhance stress tolerance.

Despite substantial progress in characterizing the functions of *ATL* genes in model plants and some crop species, the *ATL* gene family in hexaploid wheat remains largely unexplored. The inherent complexity of the wheat genome, characterized by its allohexaploid nature and substantial size, presents unique challenges for genetic investigations. However, given wheat’s global agricultural significance and its particular vulnerability to abiotic stresses, it represents a crucial target for stress tolerance research. Recent breakthroughs in wheat genomics, particularly the completion of high-quality genome sequencing projects, have provided unprecedented opportunities for the comprehensive identification and functional characterization of stress-responsive genes, including those encoding E3 ubiquitin ligases.

This study systematically identifies and characterizes the wheat *ATL* (*TaATL*) gene family, with a specific focus on investigating its potential functions in salt stress responses. This research aims to elucidate the mechanisms underlying the role of this gene family in stress adaptation in wheat. Through integrative bioinformatics approaches coupled with experimental validation, we delineate the involvement of *TaATL*s in stress response pathways and identify key regulatory nodes within this gene family. The elucidation of *ATL* gene functions in wheat will not only advance our fundamental understanding of the stress response mechanisms in this crucial crop but also provide valuable genetic markers and candidate genes for molecular breeding programs aimed at developing stress-resilient wheat cultivars. This study provides the first comprehensive genome-wide analysis of the *ATL* gene family in hexaploid wheat, revealing novel insights into their evolutionary patterns and stress-responsive expression profiles and offering valuable genetic resources for wheat improvement. In light of the escalating challenges posed by climate change and the pressing need for sustainable agricultural practices, this research is anticipated to make significant contributions to global food security initiatives.

## 2. Results

### 2.1. Genome-Wide Identification of TaATL Family Genes

**Figure 1:**
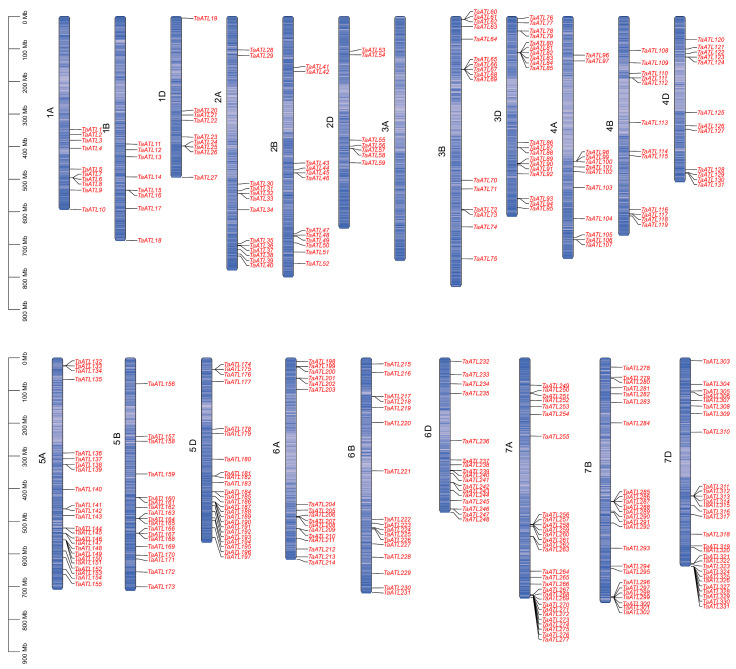
Chromosomal distribution of the Arabidopsis Tóxicos en Levadura gene family in wheat (*TaATL*). The gradient of the blue color on the chromosomes represents the gene density. The scale on the left indicates the base pair lengths (Mb) of the chromosomes.

**Figure 2:**
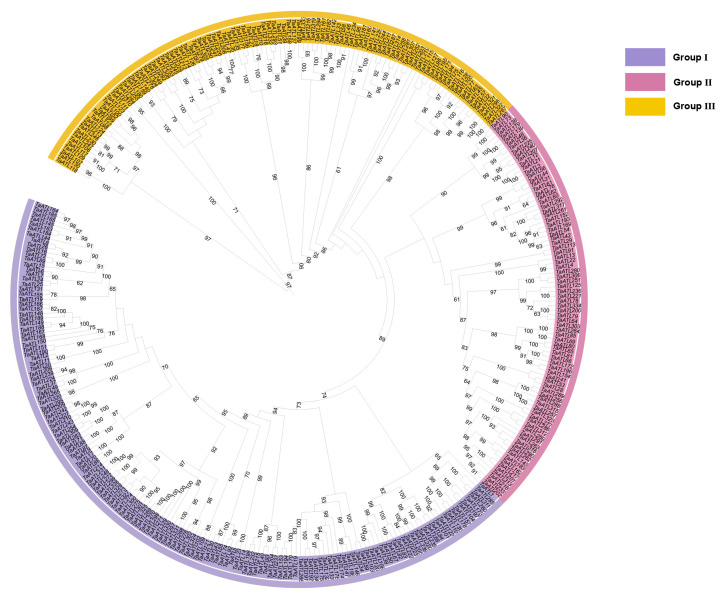
Phylogenetic analysis of *TaATL* family genes. Roman numerals I to III denote the three phylogenetic clusters. The numbers adjacent to the branches represent bootstrap values, with all values exceeding 60.

In this study, a combination of a HMMER search and BLASTp was used to identify 1198 genes of *TaATL*s. Next, domain screening was performed using the InterPro (version 104.0) platform, which resulted in the identification of 334 *ATL* family members containing either the RING-H2 finger domain or the E3 ubiquitin protein ligase domain, named *TaATL1* to *TaATL334* based on their chromosome localization (Figure 1). The *TaATL* family genes are uniformly distributed across all chromosomes except chromosome 3A, which lacks *TaATL* family genes. The RING-H2 finger and E3 ubiquitin protein ligase domains are characteristic structures of the *ATL* family E3 ubiquitin ligases, playing a crucial role in their enzymatic activity. To investigate the evolutionary relationships of the *TaATL* genes, a phylogenetic tree was constructed using the 334 genes from wheat in IQ-TREE. Based on the evolutionary tree branches and conserved structures and features of the genes, the *TaATL* family was classified into three phylogenetic clusters. Cluster I comprised 148 genes, Cluster II contained 83 genes, and Cluster III included 103 genes (Figure 2).

In addition, 105 *ATL* family genes were successfully extracted from *Aegilops tauschii*, 50 from *Triticum urartu*, 75 from *A. thaliana*, 68 from *Vitis vinifera*, and 121 from *Z. mays* (Figure S1). An ML phylogenetic tree was constructed using genes from six species, revealing six distinct evolutionary clusters, each comprising genes from all species. Among these, the *ATL* family genes of wheat exhibited the closest phylogenetic relationships with those of *A. tauschii*.

The physicochemical properties of the *TaATL* gene family were predicted, including the molecular weight, theoretical isoelectric point (pI), instability index, aliphatic index, and grand average of hydropathicity. The molecular weight varied from 13.09 kDa (*TaATL174*) to 47.85 kDa (*TaATL212*), and the theoretical isoelectric point (pI) ranged from 4.28 (*TaATL222*) to 11.1 (*TaATL220*), indicating the presence of both acidic and basic proteins in the *ATL* family. Among the proteins, 153 were acidic, 11 were neutral, and 168 were basic. The instability index spanned from 26.97 (*TaATL283*) to 95.76 (*TaATL135*), with most of the proteins exhibiting intrinsic instability. The aliphatic index ranged between 57.47 (*TaATL208*) and 121.6 (*TaATL73*). The grand average of hydropathicity varied from −0.521 (*TaATL47*) to 0.624 (*TaATL68*), with a balance between hydrophilic and hydrophobic proteins. Among them, 11 proteins were highly hydrophobic, while one protein (*TaATL242*) was highly hydrophilic. These results suggest that the *TaATL* family contains a large number of proteins with diverse characteristics. Cellular component analysis further elucidated the subcellular localization patterns, with the majority of the *TaATL* proteins predicted to be integral membrane components, while a subset was localized to intracellular organelles and nuclei (Table S1).

### 2.2. Gene Structure of TaATL Family Genes

**Figure 3:**
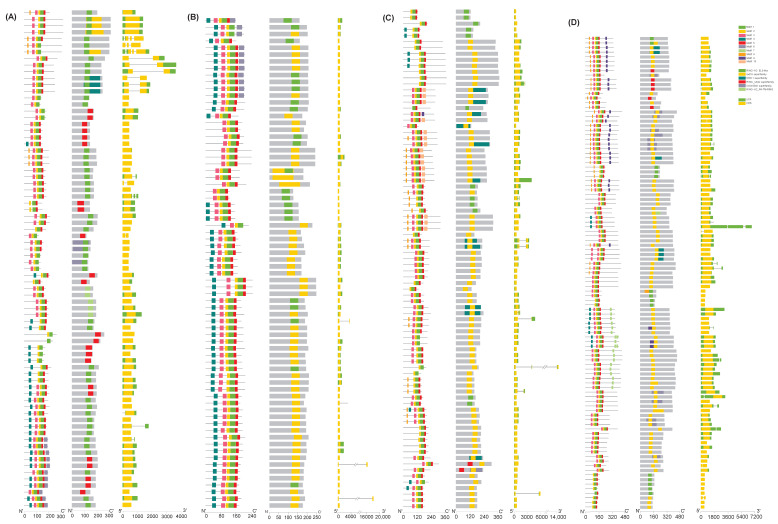
The motif distribution, conserved domains, and gene structures of *TaATL* genes. Panels (**A**,**B**) show genes from Cluster I, (**C**) from Cluster II, and (**D**) from Cluster III. In each subfigure, the first column represents the motif distribution, the second column displays the conserved domain information, and the third column illustrates the gene structure information.

Based on the wheat genome’s annotation, the gene structure diagram of the *TaATL* family members was constructed using TBtools (version 2.210) (Figure 3). Upon analyzing 334 family members, only 35 genes contained introns, indicating a very low proportion of intron-containing genes within this family. Furthermore, in the *TaATL* gene family, the majority of the genes contain both 5′-UTRs and 3′-UTRs. However, 146 genes are missing untranslated regions (UTRs) on one end, and 10 genes lack untranslated regions altogether (Figure 3). The vast majority of the genes are composed of only a single exon of approximately 500–2000 bp, with only 35 genes having two to three exons. These findings indicate that the *TaATL* gene family exhibits a highly conserved and uniform gene structure, and this is consistent with the conclusions of previous studies on *Z. mays*, *A. thaliana*, and *O. sativa*, indicating that the *TaATL* family genes are likely to function as structural genes within functional modules.

### 2.3. Conserved Domains and Motif Distribution of TaATL Family Genes

The InterPro data and phylogenetic relationship analysis demonstrated that all members of the *TaATLs* possessed one of the following domains: RING-H2, RING U-box, or the RAD18 superfamily (Figure 3). The RING finger and U-box domains represent signature structural features of E3 enzymes, and RAD18 domains have also been identified in the *ATL* gene family of *Z. mays*. Notably, the distribution of the motifs exhibited distinct clustering patterns across different phylogenetic clusters. Furthermore, substantial variations in the domain composition were observed among members of distinct evolutionary clusters. Specifically, motif4 was predominantly concentrated in phylogenetic cluster I (Figure 3A) and was scarcely present in other branches (Figure 3B). Conversely, motif9 was exclusively concentrated in phylogenetic cluster III (Figure 3C). In addition, clusters 1 and 2 predominantly lacked the RAD18 superfamily domain (Figure 3A,B), whereas the majority of cluster 3 members harbored this domain (Figure 3C). To further characterize the *TaATL* gene family, a motif analysis was performed using the MEME online tool (Figure 3D). Out of the 334 family genes analyzed, 331 contained motif1, 332 contained motif2, and 321 contained motif5. These findings highlight the intricate domain architecture and motif distribution within the *TaATL* family, suggesting potential functional diversification among its members.

### 2.4. Gene Duplication Events and Orthology Analysis of TaATL Family Genes

**Figure 4:**
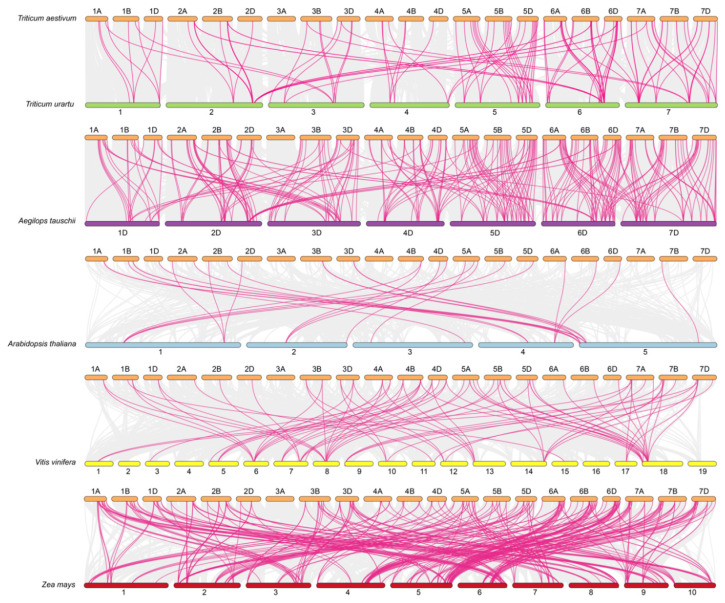
Interspecies collinearity analysis of the *TaATL* family genes and *ATL* genes in other plant species. The gray lines represent collinear blocks between the wheat genome and the other species, while the colored lines indicate collinearity among *ATL* genes.

**Figure 5:**
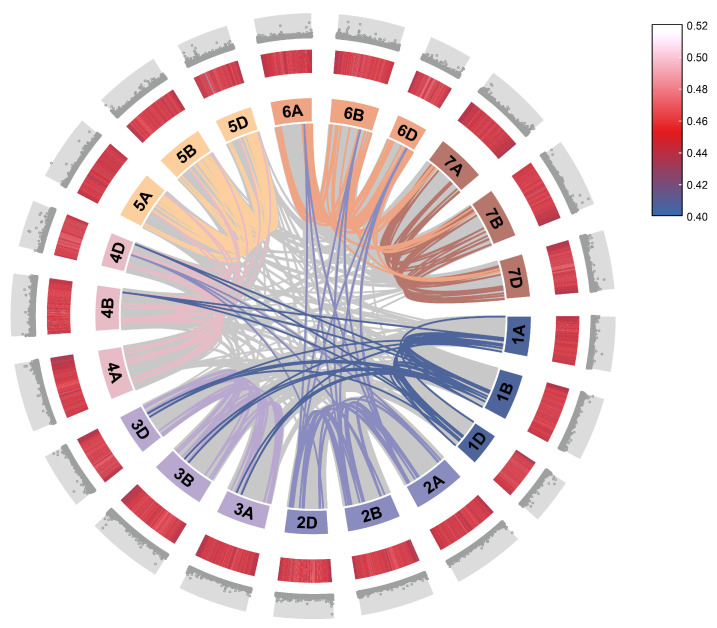
Intraspecies collinearity analysis of the *TaATL* family genes. From the outermost to the innermost layers, the figure shows the gene density on chromosomes and the GC content of the genome. The gray lines indicate collinear blocks from whole-genome hybridization in wheat, while the colored lines represents the segmental duplication of *TaATL* family genes.

To elucidate the evolutionary history and selective pressures acting on the *TaATL* gene family, we performed a selective pressure analysis and inferred gene duplication and loss events using the Notung software (version 2.9). The calculation of Ka and Ks for the *TaATL* family revealed that, with the exception of *TaATL69* and *TaATL85*, which exhibited Ka/Ks ratios greater than 1, all gene pairs displayed Ka/Ks ratios significantly smaller than 1, indicating that the majority of the *TaATL* genes are subject to strong purifying selection (Figure S2). By conducting an interspecies collinearity analysis of *TaATL* genes with two ancestral species (*T. urartu* and *A. tauschii*) and three phylogenetically related species (*A. thaliana*, *V. vinifera*, and *Z. mays*), it was found that wheat shares 91 orthologous gene pairs with *T. urartu*, 229 with *A. tauschii*, 24 with *A. thaliana*, 77 with *V. vinifera*, and 287 with *Z. mays* (Figure 4). The interspecies collinearity analysis revealed extensive collinear relationships within each chromosome set of the *TaATL* gene family. Additionally, collinear relationships were observed between chromosomes 1 and 3 and chromosomes 1 and 4, as well as between chromosomes 2 and 6 (Figure 5).

Furthermore, the evolutionary event analysis using Notung (version 2.9) demonstrated that the *TaATL* gene family underwent 50 gene loss events and 55 gene duplication events (Table S2). Across the six species analyzed, a total of 543 gene duplication events and 705 gene loss events were identified.

### 2.5. GO Analysis, Protein–Protein Interactions, and Transmembrane Domain Analysis of TaATL Family Genes

**Figure 6:**
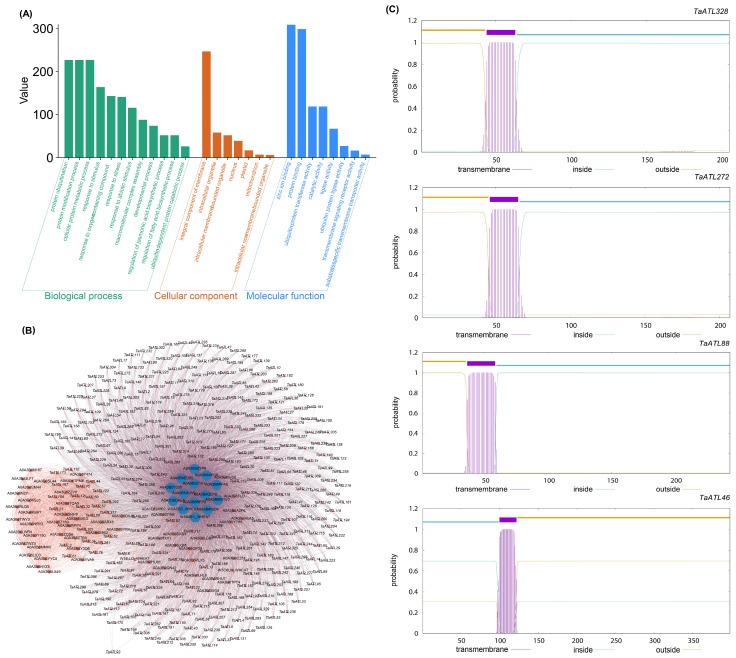
Functional enrichment, interaction analysis, and transmembrane domain analysis of *TaATL* gene family. (**A**) GO enrichment analysis of the *TaATLs*. The bar plot illustrates the functional enrichment of the *ATL* gene family in three Gene Ontology (GO) categories: biological process (BP, green bars), cellular component (CC, orange bars), and molecular function (MF, blue bars). (**B**) Protein–protein interaction (PPI) network analysis of the *TaATL*s. (**C**) Transmembrane domain analysis of *TaATL* family genes. The *y*-axis represents the probability values of amino acids in the regions corresponding to the colors.

The comprehensive functional characterization of the *TaATL* gene family was conducted through systematic GO enrichment analysis. The results of the GO analysis demonstrated the significant enrichment of the *TaATL* family genes in core biological processes, including protein metabolic, modification, and ubiquitination pathways (Figure 6A), consistent with the canonical functions of E3 ubiquitin ligases. Notably, we observed substantial enrichment in stress-responsive pathways, particularly those associated with salt stress responses (Figure 6A). The protein–protein interaction (PPI) analysis indicated that the *TaATL* family proteins interact closely with several HECT domain-containing proteins (e.g., A0A3B6QB19, A0A3B5ZYP8) and ubiquitin-like domain-containing proteins (e.g., W5ADS2_WHEAT, W5A9E1_WHEAT). Some family proteins also showed interactions with numerous RING-type domain-containing proteins. In addition, certain family proteins (e.g., *TaATL21*, *TaATL104*, *TaATL3*, *TaATL197*) formed a tight interaction network with glycosyltransferase 47 and conserved oligomeric Golgi complex subunit 8 (belonging to the COG8 family) (Figure 6B). The transmembrane domain analysis was conducted on four family proteins, namely *TaATL328*, *TaATL272*, *TaATL88*, and *TaATL46*. The results indicated that all four proteins were single-pass transmembrane proteins, with the predicted number of amino acids in their transmembrane helices exceeding 21. Furthermore, except for Ta*ATL*46, the number of amino acids in the transmembrane helices within the first 60 amino acids of these proteins was greater than 15, suggesting the potential presence of signal peptides (Figure 6C).

### 2.6. Cis-Regulatory Element Analysis of TaATL Genes

**Figure 7:**
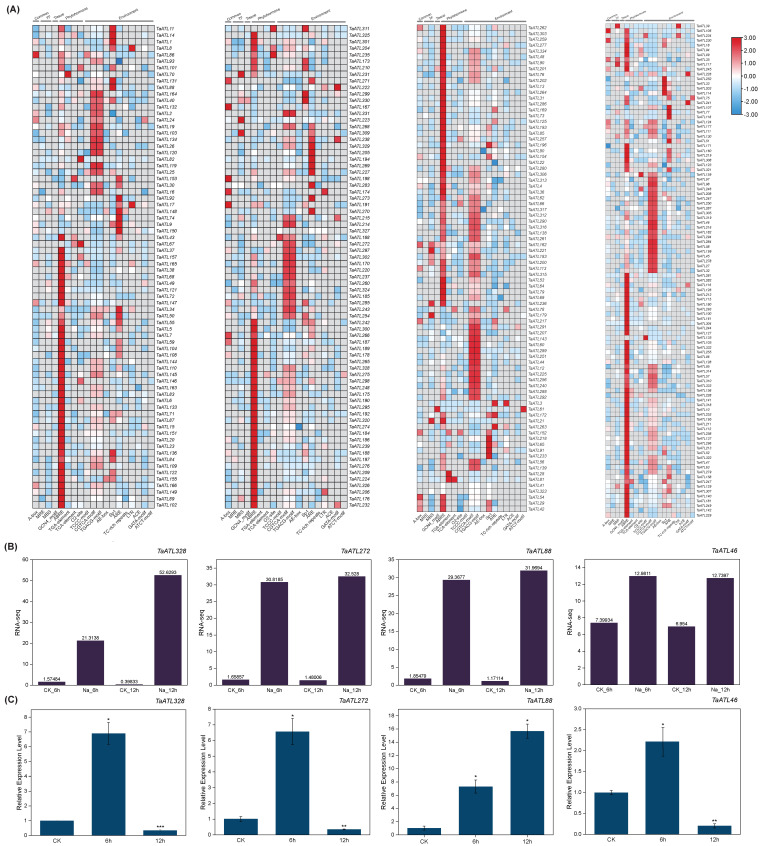
Heatmap of *cis*-regulatory elements and expression analysis of *TaATL* family genes. (**A**) Heatmap of *cis*-regulatory elements of *TaATLs*. The number of *cis*-acting elements for each gene is normalized by row. Genes in the first two figures are from Cluster 1, the third from Cluster 2, and the fourth from Cluster 3. (**B**) The RNA-seq expression levels of *TaATL328*, *TaATL272*, *TaATL88*, and *TaATL46* on Wheatomics. (**C**) The expression levels of *TaATL* family genes under salt stress. A T-test was used to determine statistically significant differences in the expression levels under different stress conditions (* *p* < 0.05, ** *p* < 0.01, *** *p* < 0.001).

To investigate the biological functions and associated signaling pathways of the *TaATL* family genes, the 2000 bp upstream sequences of the *TaATL* genes were obtained and the regulatory roles of the upstream sequence elements were analyzed. The results revealed that the promoters of the *TaATL* gene family contained a total of 70 types of elements involved in the regulation of 26 biological pathways (Figure 7A). These 70 elements are categorized into five types: common stage, transcription factors (TF), tissue, phytohormone, and environment. Among these elements, numerous *cis*-regulatory elements, such as ABA-responsive elements (ABRE), MYB-binding sites (MBS), and TCA elements, are closely related to environmental stress, regulating important stress response pathways. Notably, the promoters of the *TaATL* family members are enriched in response elements for abscisic acid (ABRE elements) and jasmonic acid (CGTCA and TGACG motifs), as well as light-responsive elements like G-box and antioxidant response elements (ARE elements).

### 2.7. Analysis of Expression Levels of TaATL Family Genes Under Salt Stress

To investigate the expression patterns of the *TaATL* family genes under salt stress, the RNA-seq data of the *TaATLs* were collected from the Wheatomics database (Figure S3). The results showed that genes in evolutionary clusters 1 and 3 exhibited significant upregulation within 48 h of salt stress treatment (e.g., *TaATL328*, *TaATL88*) (Figure 7B), while genes in evolutionary cluster 2 displayed overall low expression levels. In contrast, the majority of the genes in evolutionary cluster 3 maintained high expression levels even under non-stress conditions (e.g., *TaATL46*, *TaATL287*). This suggests that genes within this evolutionary cluster may be involved in regulating fundamental biological processes associated with ubiquitination, such as growth and development.

To further elucidate the expression patterns of the *TaATL* gene family under salt stress and validate the expression profiles of key genes under such conditions, wheat was subjected to NaCl-induced salt stress. The expression levels of *TaATL328*, *TaATL272, TaATL88*, and *TaATL46* were measured at 6 and 12 h of salinity stress (Figure 7C). The expression patterns of *TaATL328*, *TaATL272*, *TaATL88*, and *TaATL46* showed consistent trends between the RNA-seq and RT-qPCR analyses in both the control and 6 h stress treatment groups. However, in the RT-qPCR analysis, the expression levels of *TaATL328*, *TaATL272*, and *TaATL46* under 12 h stress treatment were significantly lower than those detected by RNA-seq.

The results revealed that the expression levels of *TaATL328* and *TaATL272* were significantly upregulated after 6 h of salt stress; however, their expression was markedly downregulated when the stress duration was extended to 12 h. Similarly, *TaATL46* exhibited significant downregulation in expression following 12 h of salt stress. In contrast, *TaATL88* showed substantial upregulation after 12 h of stress, with its relative expression level reaching 15-fold compared to the non-stressed control group.

## 3. Discussion

The *ATL* gene family is a subgroup of E3 ubiquitin ligases, regulating the specificity and efficiency of protein ubiquitination [7,10]. The functions and roles of the *ATL* family have been studied in other species; for example, in *A. thaliana*, *O. sativa*, and *Z. mays*, *ATL* family genes have been shown to respond to abiotic stresses [8,9]. However, the functional role of the *ATL* gene family in wheat under salt stress conditions has not been extensively studied.

Genomic characterization revealed that all identified *TaATL* family members contained either RING-H2, RING U-box, or RAD18 domains. This finding aligns with the established classification system for E3 ubiquitin ligases, which are categorized based on their characteristic domains (HECT, RING finger, or U-box), wherein the *ATL* family is specifically defined by the presence of RING finger domains [8,9,11].

The 334 *TaATL* genes exhibit a uniform distribution across wheat’s chromosomes, except for chromosome 3A, which lacks *TaATL* family genes (Figure 1). The majority of the genes are distributed at the ends of chromosomes, facilitating their transcription under stress conditions. The absence of *TaATL* genes on chromosome 3A may reflect functional specialization, where homologs on 3B and 3D have undergone subfunctionalization or neofunctionalization to compensate for the loss. This could be driven by selective pressure, particularly if these genes play roles in stress adaptation, rendering the 3A copies redundant in hexaploid wheat. Subcellular localization predictions suggest predominant membrane association, with most proteins localized to the plasma membrane and membrane-bound organelles, consistent with their classification as transmembrane integral proteins (Figure S1). This observation aligns with previous findings in *V. vinifera* and *Z. mays*, indicating that the broad distribution and transmembrane domains of the *ATL* gene family are likely essential for its membrane-associated functions [7,9]. The phylogenetic reconstruction of the *TaATL* family genes revealed three phylogenetic clusters (Figure 2). The intraspecies collinearity analysis revealed that, although previous studies have documented extensive segmental exchanges between chromosome 4 and chromosome 7 in wheat, the *TaATL* family genes remained unaffected and showed no evidence of collinearity [12] (Figure 5). The phylogenetic analysis of six species (*T. aestivum*, *T. urartu*, *V. vinifera*, *A. tauschii*, *Z. mays*, and *A. thaliana*) revealed that each phylogenetic cluster of the *ATL* gene family was composed of genes from all six species (Figure S1). This suggests that the evolution of the *ATL* gene family is relatively conserved in plants and that this family likely shares close relationships and similar functions in wheat and the other five species. The orthology analysis indicates that approximately half of the *TaATL*s show direct orthologous relationships with *T. urartu* and *A. tauschii ATL* genes, while specific *TaATL* subgroups (e.g., *TaATL262*, *TaATL291*, *TaATL290*, *TaATL316*, *TaATL263*) exhibit species-specific clustering, potentially reflecting functional specialization or adaptive evolution. Notably, the majority of these orthologous relationships were not one-to-one, indicating potential gene duplication or divergence events during evolution (Figure 4). However, the RNA-seq expression levels between gene pairs (e.g., *TaATL272* and *TaATL299*) were inconsistent, and the abundance of *cis*-regulatory elements between these gene pairs also varied (Figure 7A and Figure S3). This suggests that widespread whole-genome duplication events do not necessarily lead to gene redundancy but may instead contribute to the functional diversification of existing genes, resulting in new genes with distinct regulatory mechanisms. Of particular interest is the high degree of synteny observed between wheat and maize (*Z. mays*), which suggests a relatively close evolutionary relationship between these two species within the *ATL* gene family. Notably, despite the distant phylogenetic relationship between *A. thaliana* (dicot) and *T. aestivum* (monocot), collinear relationships were identified in the *ATL* gene family between these species, suggesting that the *ATL* genes may have been subject to strong functional constraints during evolution, leading to their high conservation. Further investigation into the evolutionary dynamics and functional implications of these orthologous relationships is warranted to elucidate the mechanisms underlying the conservation and divergence of the *ATL* gene family across these species.

The structural analysis of the *TaATL* genes reveals that 90.2% of the family members lack introns (Figure 3). This is consistent with previous findings regarding the *ATL* family in *Z. mays*, *A. thaliana*, and *O. sativa*, indicating that the *TaATL* gene family shares similarities with these species, providing evidence for *ATL* as a structural gene with functional modules, suggesting that these genes may operate with enhanced efficiency during transcription, enabling wheat to initiate stress resistance pathways more rapidly [8,9]. Furthermore, the Ka/Ks ratios of nearly all *TaATL* family genes were significantly less than 1, indicating that these genes have experienced strong negative selection pressure (Figure S2). The aforementioned results collectively demonstrate that the gene structure and arrangement within the *TaATL* family exhibit a high degree of similarity and conservation. These findings are consistent with previous studies on the *ATL* family in *A. thaliana*, *Z. mays*, and *V. vinifera*, indicating that the functional conservation of the *TaATL* family genes is highly preserved [7,8,9]. This suggests that these genes may operate with enhanced efficiency during transcription and translation, enabling them to respond rapidly to environmental stresses.

The GO enrichment analysis demonstrates the significant overrepresentation of terms related to environmental stress responses and stimulus perception, with notable enrichment on the integral components of membranes. The PPI analysis also indicated that the *TaATL* family proteins exhibit extensive interaction networks with ubiquitin ligase-related proteins, such as HECT domain-containing proteins and RING-type domain-containing proteins (Figure 6B). Furthermore, the interaction with COG8 family proteins suggests that the *TaATL* family proteins may also enhance the response to abiotic stress by modulating the Golgi apparatus’ structural reorganization through ubiquitination modifications [13]. The transmembrane domain analysis further indicated that the transmembrane regions of *TaATL* family proteins likely enable their localization to the endoplasmic reticulum (ER) (Figure 6C) [14]. As members of the E3 ubiquitin ligase family, these proteins may participate in the ER-associated degradation (ERAD) pathway by facilitating protein degradation through ubiquitination [14,15]. ERAD plays a critical role in maintaining cellular homeostasis under external stress by degrading misfolded proteins, thereby enhancing plants’ salt tolerance (Figure 6A) [16]. These functional annotations were further corroborated through a *cis*-element analysis. *Cis*-acting elements are crucial molecular switches that participate in the transcriptional regulation of dynamic gene activity networks, controlling various biological processes and enabling organisms to respond to and adapt to environmental stresses [17]. The *cis*-regulatory element analysis of *TaATL* promoters identified multiple stress-responsive motifs, including ABRE, MBS, TCA, and jasmonic acid-responsive elements (CGTCA and TGACG motifs) (Figure 7A). The prominence of ABA-responsive elements correlates with ABA’s established role as a key phytohormone in abiotic stress responses, particularly in stomatal regulation during drought and salt stress [18,19]. Osmotic stress induces ABA biosynthesis, and the ABA-responsive elements (ABREs) located upstream of the *TaATL* genes recognize ABA, thereby activating *TaATL* family gene expression and enhancing salt stress tolerance. The MBS element is the binding site for MYB transcription factors, which enhance plants’ tolerance to abiotic stimuli, such as drought stress [20,21,22,23]. Exogenous MeJA and TCA elements can also enhance plants’ tolerance to abiotic stress [24,25,26]. These findings collectively implicate *TaATL* genes in stress response regulation, potentially through the ABA and MeJA signaling pathways. Within the gene family, the core gene *TaATL52* in the protein–protein interaction network contains a larger number of ABRE elements (Figure 7A). Therefore, *TaATL*52 is strongly implicated as a regulatory gene within the *TaATL* family, playing a central role in mediating environmental stress responses. Furthermore, the significant enrichment of *TaATL* genes in the response to stimuli suggests additional involvement in biotic stress responses (Figure 7A).

The integrative analysis of the RNA-seq expression profiles reveals that four genes (*TaATL328*, *TaATL272*, *TaATL88*, and *TaATL46*) within cluster I demonstrate particularly robust stress-responsive expression profiles, strongly suggesting their regulatory roles in wheat’s salt stress adaptation mechanisms (Figure 7B). Notably, *TaATL328* exhibits pronounced upregulation as early as 6 h following salt stress induction, with sustained elevated expression levels persisting through the 24 h time point. This temporal expression pattern strongly suggests that *TaATL328* functions as a crucial early-response regulator in salt stress adaptation, likely mediating the primary stress signaling cascade through facilitating the degradation of stress-responsive repressors such as PP2C phosphatases or modulating PYR/PYL receptor–PP2C interactions to initiate downstream stress responses [27,28]. The RT-qPCR analysis further corroborated the significant upregulation of *TaATL328*, *TaATL272*, and *TaATL46* during the early phase of salt stress (Figure 7B). However, its expression level drastically declined after 12 h of salt stress, dropping below that of the non-stressed control group, inconsistent with the RNA-seq data. In contrast, *TaATL88* exhibited sustained upregulation at 12 h of salt stress, consistent with the RNA-seq findings. This distinct expression pattern suggests that *TaATL88* may participate in long-term ion homeostasis maintenance through regulating ubiquitination modifications of SOS1 or NHX-type transporters; alternatively, it may be associated with the phosphorylation of cell wall reinforcement-related enzymes such as CESA. This gene likely contributes to the activation of long-term stress adaptation mechanisms, thereby enhancing regulatory pathways during the later stages of salt stress [29,30]. Collectively, these results identify 334 highly conserved *TaATL* genes in wheat that are significantly upregulated under salt stress. Their low intron frequency and strong functional constraints further highlight their evolutionary importance in stress response mechanisms. Notably, these genes are likely involved in stress adaptation through key signaling pathways, including ABA, MeJA, and MYB.

The findings regarding the *TaATL* gene family provide crucial insights for improvements in wheat salt tolerance. Key genes such as *TaATL328*, *TaATL88*, and *TaATL46* show strong salt-responsive expression and represent promising targets for breeding. However, this study has certain limitations regarding experimental validation. The functional predictions, including the synteny analysis, subcellular localization, *cis*-regulatory elements, and protein–protein interaction networks, were primarily derived from in silico analyses. While these computational approaches provide valuable preliminary insights, they require further experimental validation to fully establish their biological relevance in plants. Future studies employing *TaATL* knockout/overexpression transgenic lines and yeast two-hybrid assays would provide direct evidence to substantiate the proposed roles of *TaATL* genes in the salt stress response.

## 4. Materials and Methods

### 4.1. Identification of TaATL Family Genes

To identify the *TaATL* genes, the genome and annotation data (*Triticum_aestivum*.IWGSC.60), along with protein sequences, were retrieved from the EnsemblPlants database (http://plants.ensembl.org/Triticum_aestivum/Info/Index (accessed on 23 November 2024)) [31]. The genome information, annotation data, and protein sequences of *T. urartu*, *A. tauschii*, *A. thaliana*, *V. vinifera*, and *Z. mays* were also obtained from the EnsemblPlants database (https://plants.ensembl.org/index.html (accessed on 21 December 2024)). The Hidden Markov Model (HMM) profiles were downloaded from the Pfam database (https://pfam.xfam.org (accessed on 23 November 2024)) [32]. The HMM profile of the *ATL* domain (PTHR45768) was acquired using the TBtools software (version 2.210) (https://github.com/CJ-Chen/TBtools-II (accessed on 16 November 2024)) [33]. The wheat genome was used as an inquiry for a BLASTp search (E-value < 1 $e^{-5}$), followed by the screening of the protein domains using InterPro (https://www.ebi.ac.uk/interpro/ (accessed on 23 November 2024)) [34]. Proteins containing RING-H2 finger and E3 ubiquitin protein ligase domains were all retained. The analysis of gene localization on chromosomes was performed using MCScanX-Super Fast within TBtools (version 2.210) [35].

### 4.2. Phylogenetic Analysis and Collinearity Analysis of TaATL Family Genes

The protein sequences were aligned using MAFFT (version 7), followed by the removal of low-quality regions with TrimAl [36,37]. A maximum likelihood (ML) phylogenetic tree was constructed using IQ-TREE2 with 1000 bootstrap replicates for ultrafast bootstrapping [38,39]. The phylogenetic tree was visualized with iTOL (version 7.0) (https://itol.embl.de (accessed on 7 January 2025)) [40].

The collinearity analysis of the *TaATLs* with *T. urartu*, *A. tauschii*, *A. thaliana*, *V. vinifera*, and *Z. mays* was conducted using MCScanX-Super Fast within TBtools (version 2.210), with sequence alignments performed by Diamond for BLAST. The phylogenetic tree of the *ATLs* from the aforementioned six species was analyzed using the TimeTree (version 5) website (https://timetree.org (accessed on 13 February 2025)) [41]. It was then visualized using the iTOL online platform (version 7.0) (https://itol.embl.de (accessed on 15 February 2025)), followed by an evolutionary event analysis performed using the Notung software (version 2.9) [42]. Additionally, a Ka/Ks (non-synonymous rate to synonymous substitution rate) selection pressure analysis and visualization were conducted using Rstudio (version 2024.12.1+563) scripts [43].

### 4.3. Analysis of cis-Acting Elements, Gene Structure, and Chromosome Location

The motifs of *TaATL* family members containing RING-H2 finger domains or E3 ubiquitin protein ligase domains were identified using MEME (version 5.5.7) (https://meme-suite.org/meme/tools/meme (accessed on 3 January 2025)) [44], with the parameter width range set to 6–50. The gene structures were obtained from the genome annotation file, while the conserved domains were acquired from the NCBI database. Additionally, the amino acid composition and hits of each motif were analyzed using the MEME online tool. The 2000 bp upstream sequences of the *TaATL* genes were extracted with TBtools (version 2.210). These sequences were subsequently submitted to the PlantCARE database (http://bioinformatics.psb.ugent.be/webtools/plantcare/html/ (accessed on 18 January 2025)) for the prediction of *cis*-acting elements [45]. The identified elements were classified and a heatmap analysis was performed using TBtools (version 2.210). The chromosome locations of the *TaATL* gene family were visualized using TBtools (version 2.210).

### 4.4. Protein–Protein Interaction, GO, and Physicochemical Property Analyses

The protein–protein interaction (PPI) analysis was performed using the STRING database (https://cn.string-db.org (accessed on 17 January 2025)) with a medium confidence score of 0.400 [46]. The Gene Ontology (GO) enrichment information for the *TaATL* gene family was obtained using the DAVID database [47]. A complete GO analysis was conducted using singular enrichment analysis (SEA) with the Yekutieli method (FDR under dependency) for multiple testing correction (significance level = 0.05). The physicochemical properties of all proteins were predicted using ExPASy online tools (https://web.expasy.org/protparam/ (accessed on 25 January 2025)) [48]. Additionally, to predict the subcellular localization of the *TaATL* gene family, the CELLO online tool (version 2.5) (https://cello.life.nctu.edu.tw (accessed on 26 January 2025)) was used [49]. Subsequently, transmembrane signals were examined using the PROTTER online tool (version 1.0) (https://wlab.ethz.ch/protter/start/ (accessed on 27 January 2025)) [50].

### 4.5. Plant Materials and Salt Stress Treatment

Plump and healthy wheat seeds were planted and germinated in a growth chamber. The chamber was maintained at a CO_2_ concentration of 400 ppm, a temperature regime of 20–22 °C, and relative humidity of 60%, with a 12 h light/12 h dark photoperiod [51]. Salt stress was induced by supplementing the hydroponic solution with 150 mM NaCl. Leaf tissue samples were collected at 6 and 12 h following stress induction.

### 4.6. Gene Expression Analysis, RNA Extraction, and Real-Time Quantitative PCR (RT-qPCR) Analysis

Protein function annotations of the *TaATL* gene family were performed using the eggNOG online tool (version 2) (http://eggnog-mapper.embl.de accessed on 12 January 2025)) [52]. The RNA-seq of the *TaATL* family genes was conducted using the Wheatomics platform (http://wheatomics.sdau.edu.cn/expression/index.html (accessed on 27 November 2024)) [53]. The transcriptome database “Transcriptome response of two wheat cultivars to salt stress” was utilized for the bioinformatics analysis. Subsequently, all genes with total expression levels (sum across all groups) exceeding 1 TPM in the RNA-seq data were selected using TBtools (version 2.210) for heatmap generation [33].

Total RNA was isolated from the leaves of *Triticum aestivum* L. using the FastPure Plant Total RNA Isolation Kit (Nanjing Vazyme Biotech Co., Ltd., Nanjing, China). cDNA synthesis was performed using the HiScript IV All-in-One Ultra RT SuperMix for qPCR (Nanjing Vazyme Biotech Co., Ltd., Nanjing, China), and gene-specific primers were designed for the four target genes using the NCBI Primer-BLAST online platform (https://www.ncbi.nlm.nih.gov/tools/primer-blast/ (accessed on 21 February 2025)) (Table S3) [54]. The RT-qPCR analysis was conducted employing the TB Green^®^ Premix Ex Taq™ II kit (Takara Bio, Kusatsu, Japan) on an ABI QuantStudio^®^ Real-Time PCR System (Applied Biosystems, Waltham, MA, USA). For the quantification of RT-qPCR, we used the Ta*Actin* gene (Table S3) as the reference gene, while each analysis included three biological replicates [55,56,57,58]. Fold changes after salt treatment were calculated as $2^{-\Delta \Delta Ct_{salt}}/2^{-\Delta \Delta Ct_{control}}$, with all procedures following the manufacturer’s protocols [59]. The qRT-PCR analysis was conducted with initial denaturation at 95 °C for 30 s, followed by 45 cycles of denaturation at 95 °C for 5 s and annealing at 60 °C for 1 min, and finalized with a series of terminal steps at 95 °C for 15 s, 60 °C for 1 min, and 95 °C for 15 s. The expression profiles of *TaATL328* and *TaATL272* were analyzed at 0 h, 6 h, and 12 h after NaCl stress treatment, while *TaATL88* and *TaATL176* were evaluated at 0 h and 12 h [59]. Each treatment group consisted of three independent biological replicates. The control group consisted of wheat plants without NaCl treatment (0 h), while the experimental groups (6 h and 12 h NaCl treatments) were both compared against this control group.

## Data Availability

The original contributions presented in this study are included in the article and Supplementary Materials.

## Supplementary Materials

The following supporting information can be downloaded at: https://www.mdpi.com/article/10.3390/plants14091306/s1, Figure S1: Gene evolutionary tree of *ATL* family genes among wheat and other species; Figure S2: Ka/Ks ratios of *TaATL* family genes; Figure S3: Heatmap of RNA-seq of *TaATL* family genes; Table S1: Physicochemical properties and subcellular localization of *TaATL* family proteins; Table S2: Gene duplication and gene loss events of the ATL family in wheat and other species; Table S3: Primer sequences of the *ATL* family. Table S4: Detailed phylogenetic analysis of the ATL gene family.

## Abbreviations

The following abbreviations are used in this manuscript:

*ATL*	*Arabidopsis Tóxicos en Levadura*
RING	Really Interesting New Gene
RAD	RING-associated domain
ABA	Abscisic acid
MYB	Myeloblastosis
ABRE	ABA-responsive element
MeJA	Methyl jasmonate
MBS	MYB-binding site
UPS	Ubiquitin–proteasome system
UTR	Untranslated region
GO	Gene Ontology
Ka	Nonsynonymous substitution rate
Ks	Synonymous substitution rate
PPI	Protein–protein interaction
