# Supplementary material for: Genome-Wide Identification of ATL Gene Family in Wheat and Their Expression Analysis in Response to Salt Stress
Source: Plants (Basel). 2025 Apr 25;14(9):1306. doi: 10.3390/plants14091306 (PMC12073748; doi:10.3390/plants14091306)
Supplement: Supplementary file 1 [file plants-14-01306-s001.zip › Supplementary materials.pdf]

plants

MDPI

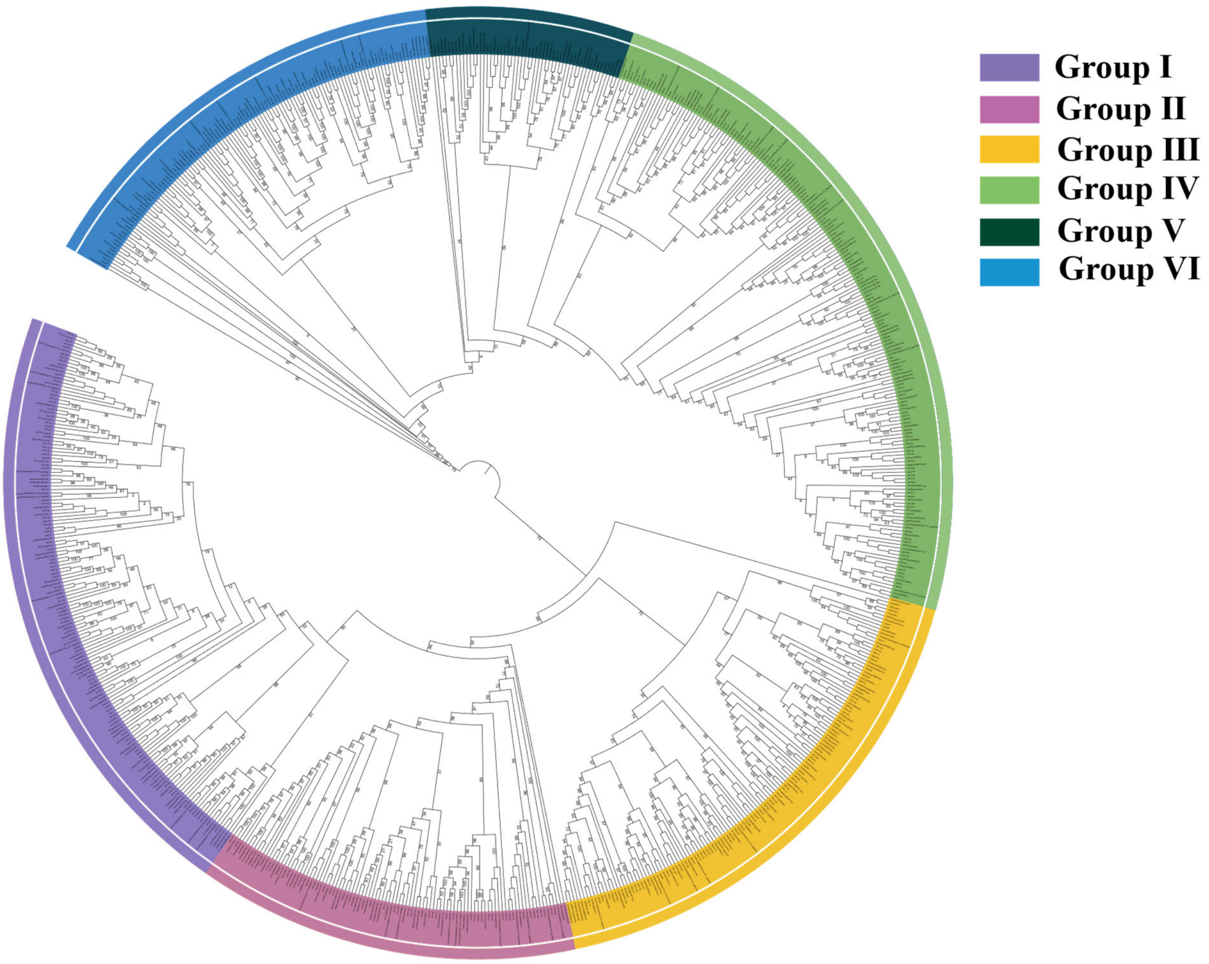

Figure S1. Gene evolutionary tree of *ATL* family genes among wheat and other species. The six different colors in the figure represent six evolutionary clusters, and the black numbers next to the branches indicate bootstrap values.

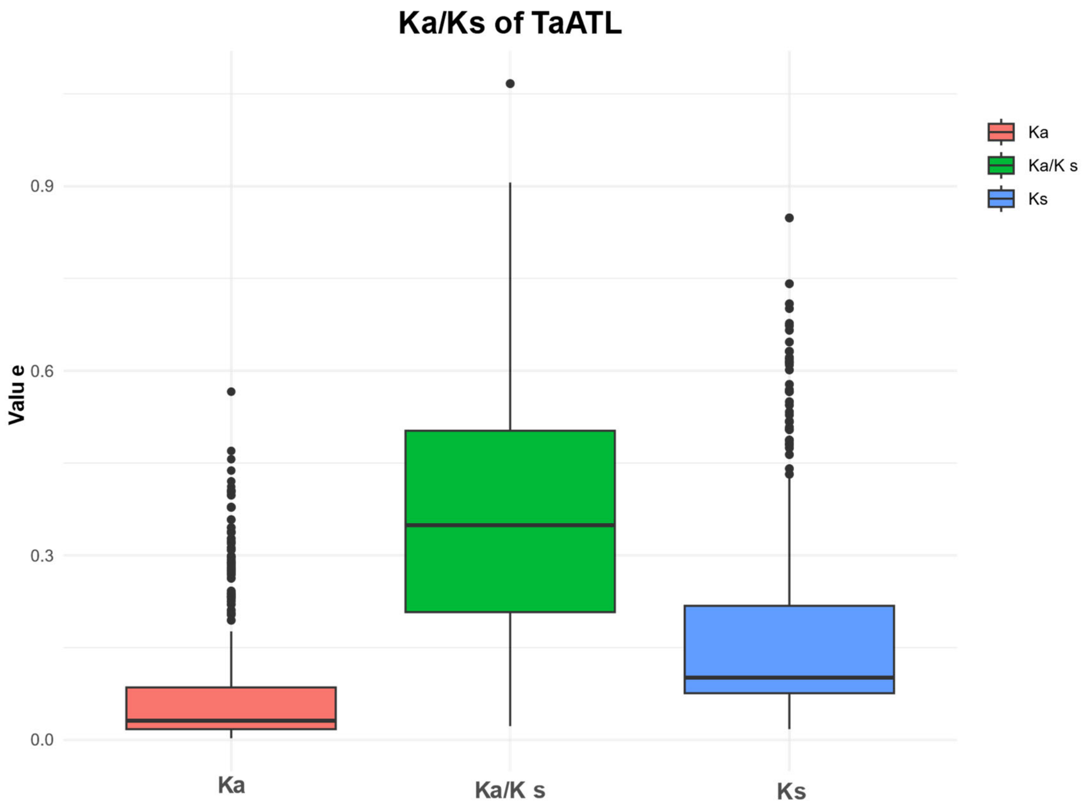

Figure S2. Ka/Ks ratios of *TaATL* family genes. The red, green, and blue boxes represent the distributions of Ka, Ka/Ks, and Ks, respectively. The upper and lower edges of each box indicate the first quartile (Q1) and third quartile (Q3), the black horizontal line represents the median, and the whiskers extend to the minimum and maximum values within 1.5 times the interquartile range (IQR). Black dots represent outliers beyond this range.

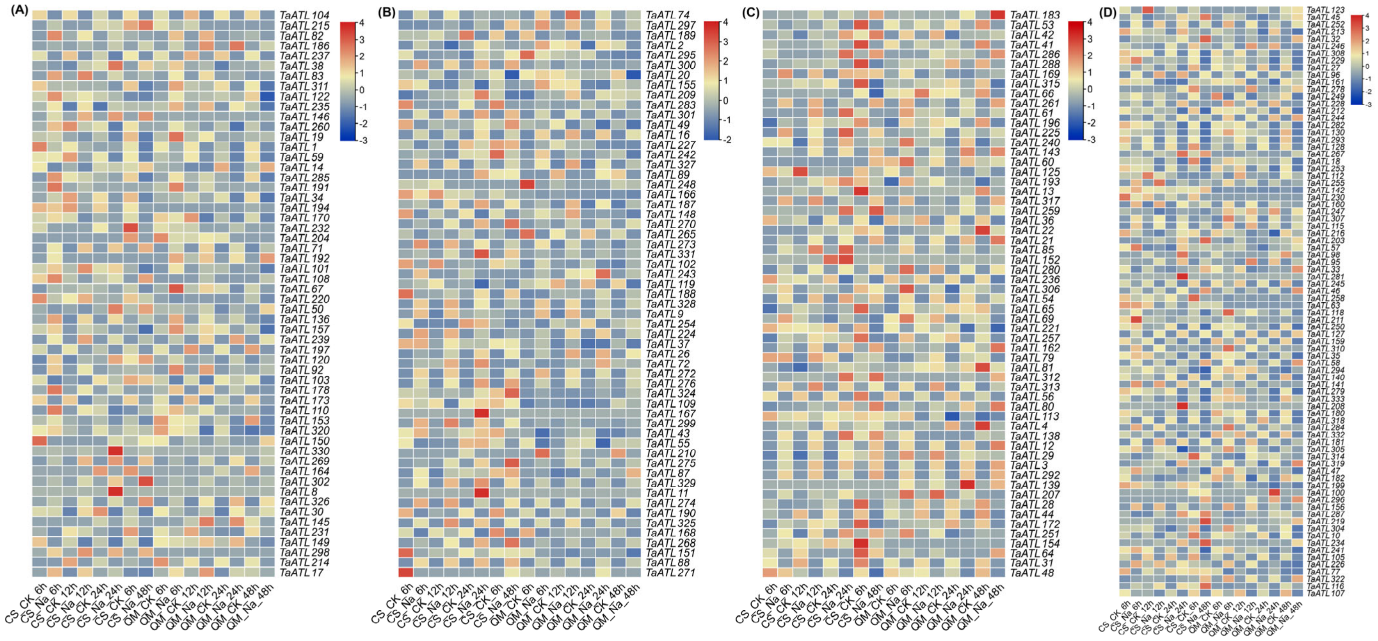

Figure S3. Heatmap of RNA-seq of *TaATL* family genes. The x-axis represents the stress duration under Chinese Spring and Qingmai 6(a salt-tolerant wheat). Gene expression levels were normalized by row, with red indicating upregulation and blue indicating downregulation. Panels (A) and (B) show genes from Cluster I, panel (C) displays genes from Cluster II, and panel (D) represents genes from Cluster III.
